# Identification of children with anaphylaxis at low risk of receiving acute inpatient therapies

**DOI:** 10.1371/journal.pone.0211949

**Published:** 2019-02-07

**Authors:** Timothy E. Dribin, Kenneth A. Michelson, Michael C. Monuteaux, Anne M. Stack, Karen S. Farbman, Lynda C. Schneider, Mark I. Neuman

**Affiliations:** 1 Division of Emergency Medicine, Cincinnati Children’s Hospital, University of Cincinnati College of Medicine, Cincinnati, Ohio, United States of America; 2 Division of Emergency Medicine, Boston Children’s Hospital, Harvard Medical School, Boston, Massachusetts, United States of America; 3 Division of Allergy and Immunology, Boston Children’s Hospital, Harvard Medical School, Boston, Massachusetts, United States of America; Universite de Bretagne Occidentale, FRANCE

## Abstract

**Objective:**

Opportunity exists to reduce unnecessary hospitalizations for children with anaphylaxis given wide variation in admission rates across U.S. emergency departments (EDs). We sought to identify children hospitalized with anaphylaxis at low risk of receiving epinephrine and other acute inpatient therapies, as these patients may be candidates for ED discharge rather than inpatient hospitalization.

**Methods:**

We conducted a single-center retrospective cohort study of children 1–21 years of age hospitalized with anaphylaxis from 2009 to 2016. Acute inpatient therapies included intramuscular (IM) or racemic epinephrine, bronchodilators, fluid boluses, vasopressors, non-invasive ventilation, or intubation. We derived age-specific (pre-verbal [<36 months] vs. verbal [≥ 36 months]) prediction rules using recursive partitioning to identify children at low risk of receiving acute inpatient therapies.

**Results:**

During the study period 665 children were hospitalized for anaphylaxis, of whom 108 (16.2%) received acute inpatient therapies. The prediction rule for patients < 36 months (no wheezing, no cardiac involvement [hypotension or wide pulse pressure]) had a sensitivity of 90.5% (CI 69.6–98.8%) and a negative predictive value of 98.3% (CI 94.1–99.8%) for identifying children at low risk of receipt of acute inpatient therapies during hospitalization. For children ≥ 36 months, the prediction rule (no wheezing, no cardiac involvement, presence of gastrointestinal symptoms) had a sensitivity of 90.8% (CI 82.7–96.0%) and a negative predictive value of 92.4% (CI 85.6–96.7%).

**Conclusions:**

We derived age specific prediction rules for children hospitalized with anaphylaxis at low risk of receiving epinephrine and other acute inpatient therapies. These children may be candidates for ED discharge rather than inpatient hospitalization.

## Introduction

Emergency department (ED) visits and hospitalizations for anaphylaxis have dramatically increased over the past decade, particularly for children [1,2]. Although most children with anaphylaxis have complete symptom resolution after initial treatment, some patients develop recurrent symptoms (biphasic or delayed reactions), or have persistent or worsening symptoms (protracted reactions) [3]. Multiple guidelines recommend observing patients presenting to the ED with anaphylaxis for 4 to 24 hours to monitor for biphasic reactions [4–7].

The lack of consensus around observation times may be attributed to wide variability in reported rates of biphasic reactions (ranging from 1% to 15%) [8,9], asymptomatic intervals of 72 hours [10], and limited information to identify children at high risk of having significant persistent or recurrent symptoms [9,11,12]. Thus, there is wide variation in hospitalization rates for children with anaphylaxis across U.S. EDs from 12% to 95% [13].

There is opportunity to standardize the ED management of children with anaphylaxis around the need for inpatient hospitalization and reduce potentially unnecessary admissions that contribute to hospital overcrowding and escalating healthcare costs. The objective of the present study was to identify children hospitalized with anaphylaxis at low risk of receiving epinephrine and other acute inpatient therapies, as these patients may be candidates for ED discharge rather than inpatient hospitalization.

## Materials and methods

We conducted a single-center retrospective cohort study of children 1–21 years of age with anaphylaxis, presenting to the ED from January 2009 to June 2016. We included a wide pediatric age range consistent with care provided at pediatric centers across the United States. The Boston Children’s Hospital institutional review board approved this study. All data were fully anonymized before they were accessed and the institutional review board waived the right for informed consent.

The study was conducted at an urban, tertiary care children’s hospital with an annual ED volume of approximately 60,000 visits. During the study period the hospitalization rate for patients presenting to the ED with anaphylaxis was 44% [14]. Children with anaphylaxis at our institution are typically observed for 4 hours prior to ED discharge and are hospitalized if they have cardiac involvement or receive 2 or more doses of IM epinephrine in the prehospital or ED settings.

We included children hospitalized from the ED with anaphylaxis. Cases were identified using *International Classification of Diseases* (ICD), *Ninth and Tenth Revision* codes of allergic reaction or adverse drug reaction (ICD-10 was implemented at our institution in October 2015), and manually reviewed to confirm the presence of anaphylaxis using the 2006 National Institute of Allergy and Infectious Disease and the Food Allergy and Anaphylaxis Network (NIAID/FAAN) diagnostic criteria for anaphylaxis (S1 Table) [15]. Children who received intramuscular (IM) epinephrine in the prehospital or ED settings were included, regardless of whether they fulfilled anaphylaxis criteria, as receipt of epinephrine may mitigate the symptoms present in the ED. Encounters fulfilling inclusion criteria were not categorized by anaphylaxis severity as the decision to hospitalize patients based on clinical severity was left to the discretion of the treating clinician.

The ICD-9 and ICD-10 codes used to identify children with anaphylaxis included: 995.0 (other anaphylactic shock), 995.1 (angioneurotic edema), 995.2 (unspecified adverse effect of drug, medicinal, and biological substance [due] to correct medicinal substance properly administered), 995.3 (allergy, unspecified), and 995.6 (anaphylactic shock due to adverse food reaction), T78.01 (anaphylactic shock due to shell fish (crustaceans), T78.02 (anaphylactic shock due to other fish), T78.03 (anaphylactic shock due to fruits and vegetables), T78.04 (anaphylactic shock due to tree nuts and seeds), T78.06 (anaphylactic shock due to milk and dairy products), T78.07 (anaphylactic shock due to eggs), T78.08 (anaphylactic shock due to other food products), T78.09 (anaphylactic shock due to unspecified food products), T78.1 (other adverse food reactions, not classified elsewhere), T78.2 (anaphylactic shock, unspecified), T78.4 (allergy, unspecified) [8,16].

We excluded children with anaphylactic reactions secondary to treatments administered in the ED, patients with a tracheostomy or receiving home respiratory support (e.g. continuous positive airway pressure [CPAP] or bilevel positive airway pressure [BiPAP]) as well as children with hereditary angioedema, mast cell activation disorders, and somatoform disorder.

We abstracted variables from the electronic medical record, including patient demographics, prior history of anaphylaxis or asthma, and reaction characteristics (time and type of allergen exposure). Encounters in which there was uncertainty around the specific trigger, or if the trigger was not documented were classified as having an “unknown” trigger. We also collected information regarding symptoms reported prior to ED arrival, and medications administered in the prehospital setting (intramuscular [IM] epinephrine, inhaled beta agonists, and intravenous [IV] fluid boluses). Lastly, vital signs and physical examination findings from the initial ED evaluation were obtained. Symptoms and examination findings were categorized by organ system involvement (mucosal/dermatologic [generalized hives, pruritus or flushing, swollen lips, tongue, or uvula], respiratory [dyspnea, wheezing, stridor, hypoxemia], cardiac [hypotension, hypotonia, syncope, incontinence], gastrointestinal [abdominal pain, vomiting]) in accordance with NIAID/FAAN criteria [15]. Timestamps and medication administrations were obtained through an automated query of the electronic medical record, and historical/examination findings were obtained through manual record review. Historical and physical examination findings, as well as prehospital treatments were categorized as present/received or absent/not received based on documentation in the electronic medical record. Variables not recorded in the record were categorized as absent/not received.

Our primary outcome was receipt of acute inpatient therapies during hospitalization. We used an inclusive definition of acute inpatient therapies to avoid miscategorizing patients who required hospitalization. Acute inpatient therapies included inhaled beta agonists, epinephrine (parenteral or racemic), magnesium sulfate, terbutaline, IV fluid boluses, vasopressors, non-invasive ventilation, intubation, or central line placement. Antihistamines and corticosteroids were not considered acute inpatient therapies as they can routinely be administered in the outpatient setting, and would not typically constitute therapies requiring hospitalization. Therapies received in the ED after 4 hours were categorized as inpatient treatments, given our standard observation period prior to ED discharge is 4 hours, and to be as inclusive as possible of worrisome outcomes.

We applied the TRIPOD guidelines for deriving and reporting prediction models [17]. Recursive partitioning analysis was used to identify children at low risk of receiving acute inpatient therapies. Two age-based models were derived (preverbal [less than 36 months] and verbal [36 months or greater]) based on the ability to verbally communicate symptoms associated with anaphylaxis (e.g. nausea, abdominal pain).

Candidate predictors included information from the prehospital setting and available to the clinician at the time of initial ED evaluation (symptoms, vital signs, and physical examination findings). Treatments received in the ED were not included as predictors because we wanted to create models to facilitate early decision making around the need for inpatient hospitalization. A priori, we restricted the number of candidate predictors to variables 1) previously shown to be associated with the risk of biphasic anaphylaxis, protracted or refractory anaphylaxis, and receipt of 2 or more doses of epinephrine [3,8–11,18–31]; and, 2) considered clinically relevant based on consensus from the authors. Candidate predictors included unknown trigger, history of anaphylaxis or asthma, cardiac involvement (hypotension, hypotonia, syncope, incontinence, wide pulse pressure), gastrointestinal involvement (nausea, abdominal pain, emesis, diarrhea), respiratory distress (dyspnea, retractions), cough, wheezing, hypoxia (O_2_ saturation ≤ 92%) [32], stridor, and receipt of beta agonists or IM epinephrine. Cardiac involvement was defined by NIAID/FAAN criteria and expanded to include wide pulse pressure (defined as diastolic blood pressure lower than or equal to half the systolic blood pressure) which has been associated with an increased risk of biphasic anaphylaxis [8,15]. The association or interaction among symptoms and physical examination findings were not assessed as we sought to identify the predictive value of individual covariates for the outcome.

Because the goal of the analyses was to identify children at very low risk of receiving acute inpatient therapies, we sought to maximize the sensitivity and negative predictive value of the models. The decision tools were derived using binary recursive partitioning as implemented in the JMP Pro software package (JMP Pro 12.0.1). The logworth statistic (defined as *-log(p value)*) was used to select the optimal candidate predictor at each node. The candidate predictor with the largest logworth statistic was chosen [33]. After the initial split, the branch of the tree displaying the greater negative predictive value was selected for additional splitting. This process was repeated until the terminal low-risk node reached a negative predictive value of ≥95% or until the sample size of the low risk group in that node dropped below 100. We categorized a child at very low risk of receiving acute inpatient therapies if none of the predictors identified by the model were present. We reported test characteristics for the decision rules derived from the recursive partitioning models and calculated exact (also known as Clopper-Pearson) [34] binomial 95% confidence intervals (CI). We evaluated the discrimination of the models using the area under the curve (AUC) from a receiver operating characteristic analysis of the models. We assessed the calibration of the models using the Hosmer-Lemeshow goodness-of-fit test, which evaluates whether there is a difference between the observed and predicted outcomes; a statistically significant result indicates poor model fit. To evaluate the internal validation of our results, we conducted a five-fold cross validation procedure for each of the two age-specific models [35]. In this procedure, the dataset is randomly divided into five equally sized partitions. Each partition is in turn set aside (i.e., the hold-out partition) while the model is estimated using the aggregate of the remaining four partitions. The estimates derived in the aggregate subset are then applied to the hold-out partition and classification accuracy is determined. The results of these five calculations are combined to arrive at a cross-validated AUC and overall correct classification proportion, which were compared to the corresponding values from the derivation model. Model evaluation and validation statistics were calculated using Stata (Stata Statistical Software, Release 14.1, StataCorp, College Station, TX) [36].

## Results

**Fig 1:**
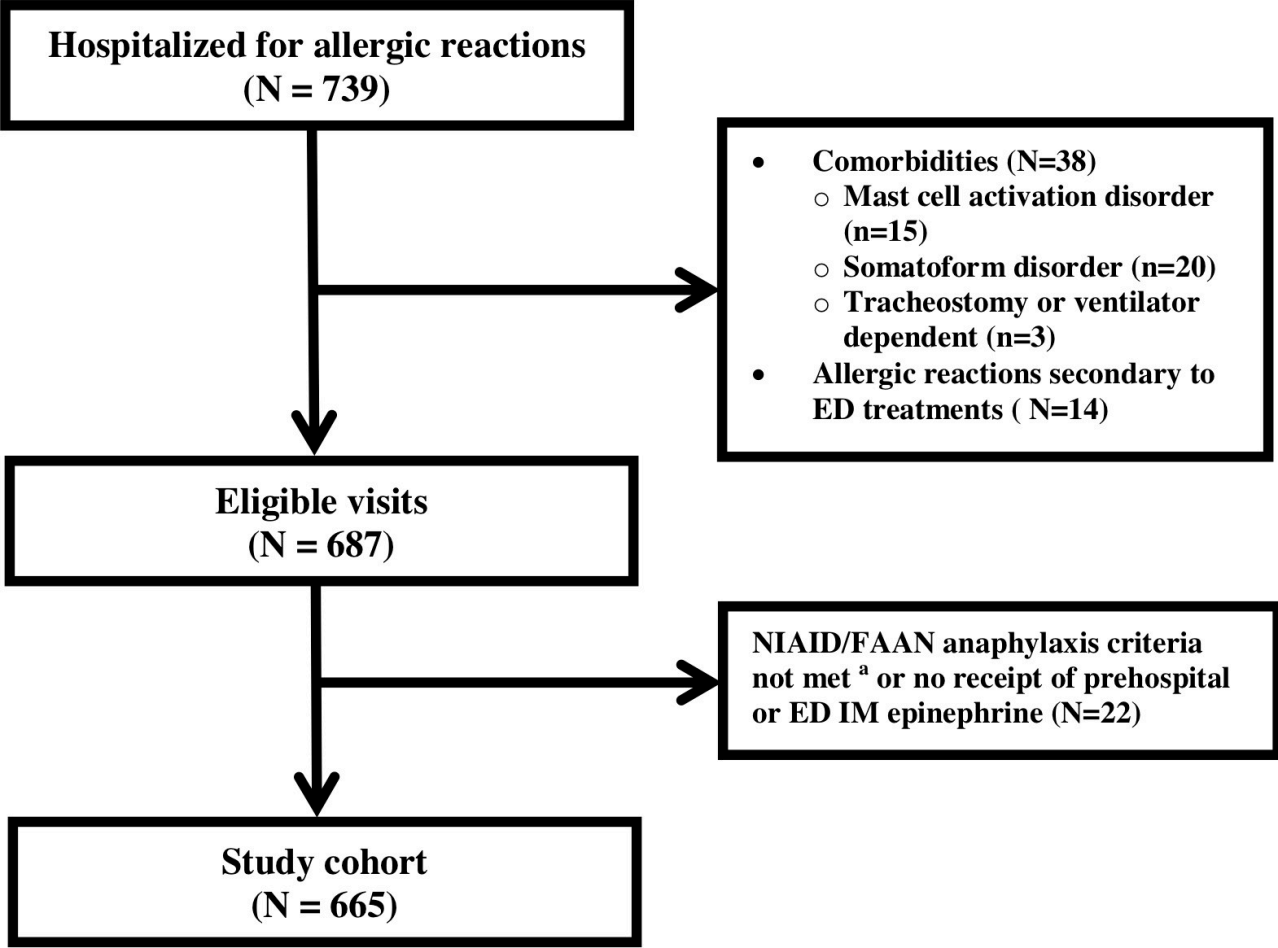
Patient flow diagram. ^a^ Manual chart review performed applying the 2006 National Institute of Allergy and Infectious Disease and the Food Allergy and Anaphylaxis Network diagnostic criteria for anaphylaxis [15].

After exclusions and manual review using NIAID/FAAN anaphylaxis criteria, 665 children hospitalized for anaphylaxis were included in the study, of which 16 (2.4%) had a preceding ED visit for anaphylaxis within 72 hours (Fig 1). A total of 595 children (89.5%) were admitted to a general inpatient floor and 70 (10.5%) to the step-down unit or an ICU. Five children (0.8%) were transferred from the floor to an ICU during hospitalization and the median inpatient length of stay was 18 hours (IQR 14–21 hours). Following discharge from an inpatient hospitalization for anaphylaxis, subsequent ED visits for anaphylaxis within 72 hours occurred in 1.4% (n = 9) of children, with 0.8% (n = 5) leading to hospitalization.

Patient characteristics are presented in Table 1. A total of 31.7% (n = 211) of children were less than 36 months of age, with a male predominance of 56.7% (n = 377). Food was the most common trigger (n = 389, 58.5%), however, the trigger was unknown in 219 (32.9%) patients. In the prehospital setting, 258 patients (38.8%) received a single dose of epinephrine, 113 (17%) received beta agonists, and 81 (12.2%) received 2 or more doses of IM epinephrine. A total of 108 children (16.2%) received acute inpatient therapies during hospitalization, including treatments received after 4 hours of ED care. The most common therapies were beta agonists (n = 72, 10.8%), IV fluid boluses (n = 25, 3.8%), and IM epinephrine (n = 18, 2.7%); 15 patients received a single dose of epinephrine and 3 received 2 or more doses. One patient (0.2%) required non-invasive ventilation. (Table 2)

**Table 1:** Characteristics of children hospitalized with anaphylaxis.

Characteristics	Age <36 months N (%) n = 211	Age ≥ 36 months N (%) n = 454
***Patient demographics***		
Male	146 (69.2)	231 (50.9)
Race		
White	87 (43.1)	194 (45.8)
Black	37 (18.3)	101 (23.8)
Other	78 (38.6)	129 (30.4)
Ethnicity		
Hispanic	47 (25.4)	73 (18.4)
Language		
English	182 (91.5)	388 (88.6)
***Past medical history***		
Asthma	44 (20.9)	223 (49.1)
Anaphylaxis	37 (17.5)	148 (32.6)
***Reaction trigger***		
Food	130 (61.6)	259 (57.0)
Medication	6 (2.8)	36 (7.9)
Venom	1 (0.5)	7 (1.5)
Other	1 (0.5)	6 (1.3)
Unknown	73 (34.6)	146 (32.2)
***Clinical manifestations of anaphylaxis*** ^a^		
Respiratory	171 (81.0)	402 (88.5)
Cardiac	18 (18.5)	68 (15.0)
Gastrointestinal	116 (55.0)	232 (51.1)
Dermatologic or mucosal	208 (98.6)	426 (93.8)
***Prehospital therapies***		
IM epinephrine doses		
None	116 (55.0)	210 (46.3)
1	75 (35.5)	183 (40.3)
2 or more	20 (9.5)	61 (13.4)
Beta agonist	27 (12.8)	86 (18.9)
IV Fluid bolus	9 (4.3)	28 (6.2)
***Transport from another facility***	29 (14.0)	77 (17.3)
***Prehospital and ED vitals***		
Hypoxia ^b^	4 (1.9)	13 (2.9)
Hypotension ^c^	3 (1.5)	6 (1.3)
Wide pulse pressure ^d^	25 (12.3)	67 (14.9)

^a^ Categories are not mutually exclusive

^b^ Hypoxia defined as O_2_ saturation ≤92% [32]

^c^ Defined according to Pediatric Advanced Life Support guidelines: systolic blood pressure <70 mmHg for 1 to 12 months of age, <70 mmHG + (age in years x 2) for 1 to 10 years, and <90 mmHg for older than 10 years [37]

^d^ Defined as a diastolic blood pressure lower than or equal to half the systolic blood pressure [37]

**Table 2:** Receipt of acute inpatient therapies among children hospitalized with anaphylaxis.

Characteristics	N (%) n = 665
**Any acute inpatient therapy** Epinephrine, parenteral Single dose, intramuscular Two or more doses, intramuscular Epinephrine, intravenous drip Inhaled beta agonist Intravenous fluid bolus Racemic epinephrine Vasopressors (dopamine, norepinephrine) Non-invasive ventilation	**108 (16.2)**^†^ 29 (4.4) 15 (2.3) 3 (0.5) 11 (1.7) 72 (10.8) 25 (3.8) 5 (0.8) 1 (0.2) 1 (0.2)
Mechanical ventilation	0 (0)

^†^ Treatments not mutually exclusive

**Fig 2:**
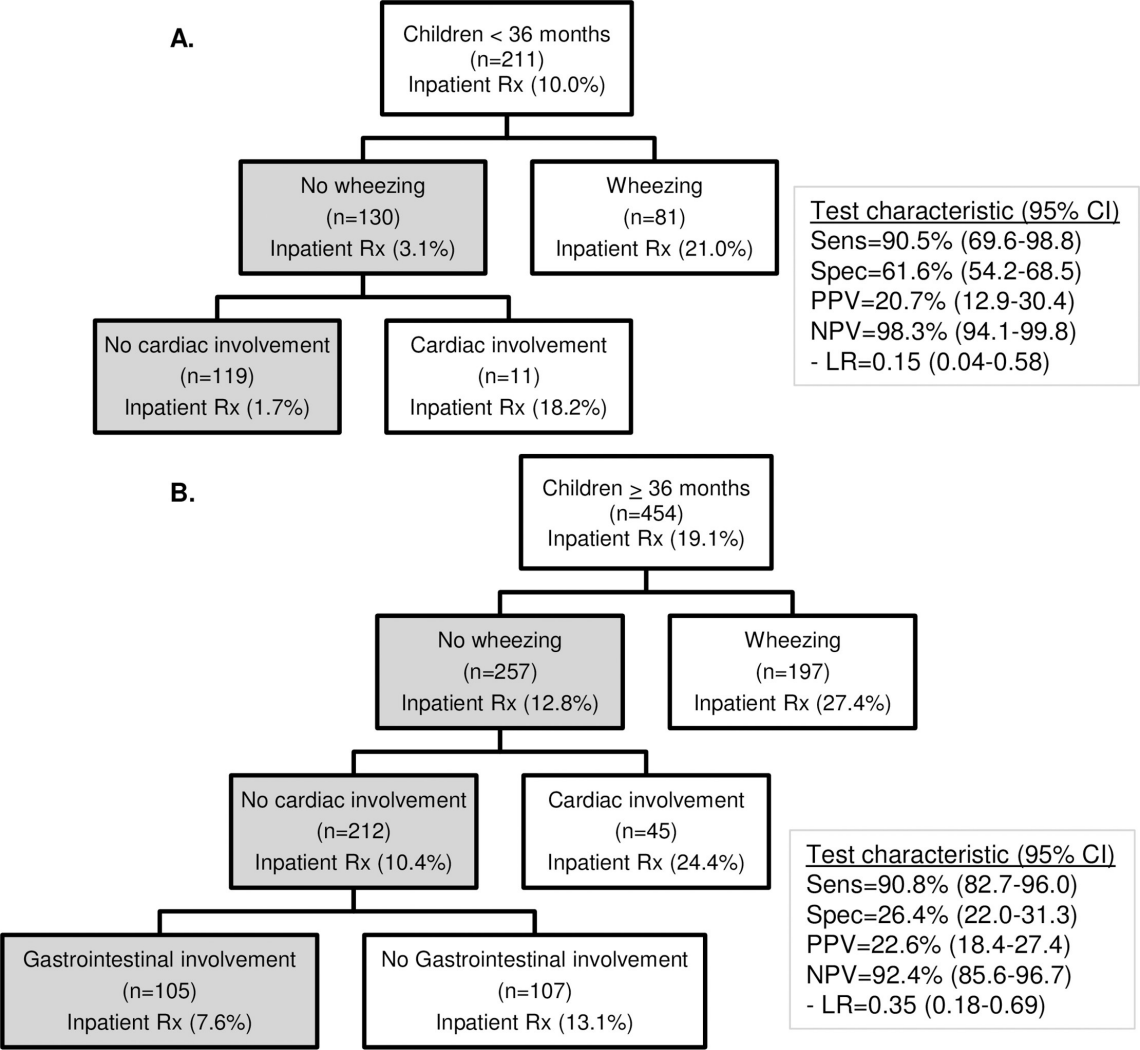
Prediction tree for acute inpatient therapies in children < 36 months (A) and ≥ 36 months (B).

A total of 557 children (83.8%) did not receive acute inpatient therapies during hospitalization. For preverbal children, characteristics associated with not receiving acute inpatient therapies during hospitalization included: 1) no wheezing, and 2) no cardiac involvement. Absence of those two predictors had a sensitivity of 90.5% (95% CI 69.6–98.8%) and negative predictive value of 98.3% (95% CI 94.1–99.8%) for the outcome of receiving acute inpatient therapy (Fig 2). The AUC for the model was 0.77 (95% CI 0.69–0.85) and the calibration test did not detect evidence of poor fit (χ^2^_(4)_ = 2.59, p = 0.11). From the cross validation procedure, the AUC was 0.68 (95% CI 0.56–0.80) and the correct classification rate was 65.9% compared to 64.5% in the derivation model.

Among verbal children, characteristics associated with not receiving acute inpatient therapies during hospitalization included: 1) no wheezing, 2) no cardiac involvement, and 3) presence of gastrointestinal involvement. Absence of these three predictors had a sensitivity of 90.8% (95% CI 82.7–96.0%) and negative predictive value of 92.4% (95% CI 85.6–96.7%). The AUC for the model was 0.70 (95% CI 0.64–0.76) and the calibration test was not significant (χ^2^_(4)_ = 1.54, p = 0.82). From the cross validation procedure, the AUC was 0.68 (95% CI 0.62–0.75) and the correct classification rate was 44.9% compared to 38.8% in the derivation model.

Two preverbal patients were misclassified by the models as low risk; both received inpatient IV fluid boluses for dehydration and neither received IM epinephrine. Among the eight verbal children misclassified as low risk, four received a single dose of IM epinephrine after 4 hours of ED care. Of these children, three had subjective symptoms of throat tightness or abdominal pain and one became hypotensive shortly after having normal vital signs in triage and was admitted to an ICU for hemodynamic instability requiring fluid resuscitation.

## Discussion

We identified children hospitalized with anaphylaxis at low risk of receiving acute inpatient therapies. The models are simple and incorporate predictors (lack of wheezing, no cardiac involvement, and presence of gastrointestinal involvement) that are intuitive in clinical practice and readily available to clinicians at the time of initial ED evaluation. Over 50% of children younger than 3 years of age and 20% of older children were classified as low-risk for receiving acute inpatient therapies. If validated in future prospective studies, these findings may be incorporated into guidelines designed to reduce pediatric hospitalizations for anaphylaxis.

During the past decade, ED visits for anaphylaxis among children and adults have increased by 101% and hospitalizations by 37.6% [1,2]. Over a 4-year period from 2009 to 2013, ED visits doubled across 35 U.S. children’s hospitals from 5.7 to 11.7 patients per 10,000 ED visits with significant variation in hospitalization rates (12%-95%) [13]. A decision support tool that accurately identifies patients at low risk of receiving acute inpatient therapies may help standardize the ED management of patients with anaphylaxis around the need for admission. Identification of low-risk patients who may be candidates for ED discharge rather than hospitalization may reduce unnecessary admissions that are difficult for families and contribute to hospital overcrowding and escalating healthcare costs.

Our findings demonstrate that among children with anaphylaxis, patients with gastrointestinal and dermatologic/mucosal involvement who do not have wheezing or cardiac involvement are unlikely to receive acute inpatient therapies. Similar to previous investigations, we observed that the presence of wheezing and cardiac involvement, including hypotension and wide pulse pressure, were associated with receipt of acute inpatient therapies during hospitalization. In an observational study of children and adults, wheezing was a significant predictor of biphasic anaphylaxis (odds ratio [OR] 2.6) [10]; and in another large pediatric cohort study, children who received beta agonists were twice as likely to develop biphasic anaphylaxis compared to those who did not receive this medication [8]. The same study found wide pulse pressure to be an independent predictor of biphasic reactions (OR 2.9) [8], likewise, hypotension was a risk factor for biphasic anaphylaxis in a meta-analysis of pediatric and adult patients (OR 2.2) [11]. In prior studies, the presence of gastrointestinal symptoms has been associated with biphasic anaphylaxis [10]; however, we observed that the presence of gastrointestinal symptoms among verbal children without wheezing and cardiac involvement was associated with a reduced risk of receiving acute inpatient therapies. We suspect patients hospitalized primarily for the presence of gastrointestinal symptoms (and without cardiac involvement or wheezing) have less severe reactions than those patients meeting other anaphylaxis criteria. In the absence of wheezing or cardiac involvement, few children in our study received acute inpatient therapies (1.7% of children <36 months, and 10.4% for older children).

This study is subject to limitations, mostly related to the retrospective and single-center design. Similar to other studies in this area, there was likely considerable variation in hospitalization rates among clinicians caring for children with anaphylaxis over the course of the study. Furthermore, patients may have had symptoms and physical examination findings not documented in the electronic medical record. Similarly, symptoms and examination findings may not reflect patient acuity upon ED arrival if documentation occurred after receipt of therapies that mitigated reaction severity. The proportion of children classified as having an “unknown” allergen trigger was higher than reported in other studies, which may reflect regional epidemiologic variation or because of our broad definition of unknown trigger [8,19].

An additional set of limitations relates to the classification of inpatient therapies. We were unable to discern whether inpatient medications such as albuterol were administered for treatment of anaphylaxis or for co-existing asthma, which was prevalent in our study cohort. Additionally, patients may have received intravenous fluid for dehydration and not hemodynamic instability from anaphylaxis. We used a conservative definition of acute inpatient therapies based on group consensus to avoid misclassifying patients who required hospitalization. Furthermore, hospitalized patients may have had recurrent or persistent anaphylactic symptoms after ED care that were under-recognized and treated. We suspect this was an uncommon event given the fact that 0.8% of patients discharged from the inpatient setting had subsequent ED visits for anaphylaxis resulting in hospitalization.

The study was conducted in a tertiary care pediatric institution and limited to hospitalized children which may limit the generalizability of our findings. It should be noted that our hospitalization and ICU admission rates for patients with anaphylaxis are similar to other U.S. children’s hospitals [13,14]. We studied hospitalized children because our goal was to reduce unnecessary hospitalizations; however, we were unable to assess whether children discharged from the ED received acute therapies such as epinephrine after discharge. We suspect these were rare events given our low ED revisit rate for anaphylaxis (2.1%) [14] and because none of the children in our cohort who had a preceding ED visit for anaphylaxis (n = 16, 2.4%) received IM epinephrine during hospitalization.

Before these findings can be applied in clinical practice, they require prospective validation with close attention to the outpatient course of discharged patients. Low risk children (even patients who receive repeat epinephrine) may be candidates for ED discharge rather than inpatient hospitalization, thus avoiding unnecessary hospitalizations that contribute to hospital overcrowding, escalating healthcare costs, and the unmeasured financial and emotional burden placed on patients and families. Similar to other clinical decision support tools, these findings are intended to assist and not replace clinical judgement. Even among low risk patients, clinicians may decide to hospitalize children based on information not included in the models (e.g. comorbidities, ease of outpatient follow-up, access to epinephrine auto-injectors at discharge, family comfort). Similarly, patients categorized as low risk who are discharged from the ED may be at risk of experiencing recurrent symptoms; thus, most patients should be observed in the ED prior to discharge to monitor for recurrent reactions, ensure access and teaching around epinephrine auto-injectors, and to facilitate outpatient follow up [38].

## Conclusions

We identified characteristics of children hospitalized with anaphylaxis at low risk of receiving epinephrine and other acute inpatient therapies. These patients may be candidates for ED discharge rather than inpatient hospitalization, and, if validated in prospective studies application of the models may help reduce hospital overcrowding and healthcare spending.

## Supporting information

S1 Table Clinical criteria for diagnosing anaphylaxis [15]. (PDF)
