# Supplementary material for: Identification of children with anaphylaxis at low risk of receiving acute inpatient therapies
Source: PLoS One. 2019 Feb 7;14(2):e0211949. doi: 10.1371/journal.pone.0211949 (PMC6366886; doi:10.1371/journal.pone.0211949)
Supplement: S1 Table — (PDF) [file pone.0211949.s001.pdf]

S1 Table. Clinical criteria for diagnosing anaphylaxis [15]

Anaphylaxis is highly likely when any one of the following 3 criteria are fulfilled:

1. Acute onset of an illness (minutes to several hours) with involvement of the skin, mucosal tissue, or both (eg, generalized hives, pruritus or flushing, swollen lips-tongue-uvula)
   AND AT LEAST ONE OF THE FOLLOWING

   a. Respiratory compromise (e.g. dyspnea, wheeze-bronchospasm, stridor, reduced peak expiratory flow, hypoxemia)
   b. Reduced blood pressure or associated symptoms of end-organ dysfunction (e.g. hypotonia [collapse], syncope, incontinence)
2. Two or more of the following that occur rapidly after exposure to a likely allergen for that patient (minutes to several hours):
   a. Involvement of the skin-mucosal tissue (e.g. generalized hives, itch-flush, swollen lips-tongue-uvula)
   b. Respiratory compromise (e.g. dyspnea, wheeze-bronchospasm, stridor, reduced peak expiratory flow, hypoxemia)
   c. Reduced blood pressure or associated symptoms (e.g. hypotonia [collapse], syncope, incontinence)
   d. Persistent gastrointestinal symptoms (e.g. crampy abdominal pain, vomiting)
3. Reduced BP after exposure to known allergen for that patient (minutes to several hours):
   a. Infants and children: low systolic BP (age specific) or greater than 30% decrease in systolic BP[†]
   b. Adults: systolic BP of less than 90 mm Hg or greater than 30% decrease from that person's baseline

---

[†]Low systolic blood pressure for children is defined as less than 70 mm Hg from 1 month to 1 year, less than (70 mm Hg + [2 x age]) from 1 to 10 years, and less than 90 mm Hg from 11 to 17 years
